# miR-204-5p attenuates inflammatory infiltration in early-stage chronic renal allograft dysfunction by modulating the IL-11/ERK1/2-NF-κB axis

**DOI:** 10.3389/fimmu.2026.1764404

**Published:** 2026-08-11

**Authors:** Lin Wang, Lin Zhu, Sai Peng, Qiang Zheng, Qinghua Zhang, Yixin Chen, Yi Lu, Jin Deng

**Affiliations:** 1 Department of Nephrology, The First Affiliated Hospital of University of South China, Hengyang, China; 2 Department of Anesthesiology, The First Affiliated Hospital, Hengyang Medical School, University of South China, Hengyang, China; 3 Department of Emergency, Sichuan Provincial People’s Hospital, University of Electronic Science and Technology of China, Chengdu, Sichuan, China; 4 The Health Management Center, The First Affiliated Hospital, Hengyang Medical School, University of South China, Hengyang, China

**Keywords:** chronic allograft dysfunction, IL-11, inflammation, kidney transplant, miRNA

## Abstract

**Background:**

Chronic renal allograft dysfunction (CRAD) continues to limit the long-term survival of transplanted kidneys. Inflammatory cell accumulation during the early phase of CRAD may accelerate fibrotic remodeling and graft dysfunction, although the regulatory mechanisms driving this process have not been fully clarified. Interleukin-11 (IL-11) is increasingly recognized as a fibro-inflammatory mediator, but whether miRNA-dependent regulation of IL-11 participates in early CRAD-associated inflammation remains unknown. This study aimed to investigate whether miR-204-5p regulates early inflammatory injury in CRAD by targeting IL-11.

**Methods and results:**

A rat CRAD model was established by orthotopic renal transplantation, and kidney tissues were collected at 12 weeks post-transplantation to assess early inflammatory and fibrotic changes. IL-11 expression was inhibited *in vivo* using IL-11-shRNA AAV, and miR-204-5p was overexpressed using mmu-miR-204-5p AAV. Histological staining, immunohistochemistry, RT-qPCR, ELISA, and renal function analyses were performed. *In vitro*, BUMPT cells were stimulated with TGF-β1 to mimic CRAD-associated early inflammatory and pro-fibrotic responses. IL-11/IL-11RA signaling was blocked using neutralizing antibodies or siRNA. The ERK1/2 inhibitor PD98059 was used to assess downstream pathway activation. The direct interaction between miR-204-5p and IL-11 was validated by dual-luciferase reporter assays, and rescue experiments with recombinant IL-11 were performed to determine whether IL-11 mediates the effect of miR-204-5p.

**Conclusion:**

miR-204-5p attenuates early inflammatory injury in CRAD by directly targeting IL-11 and suppressing IL-11-associated ERK1/2-NF-κB inflammatory signaling. The miR-204-5p/IL-11 axis may represent a potential regulatory pathway and therapeutic target for early inflammatory injury in CRAD.

## 1 Introduction

End-stage renal disease (ESRD) has emerged as a global health issue, with the number of cases increasing steadily. Data from the United States Renal Data System showed that 124,500 incident ESRD cases were recorded in 2017, while the prevalent ESRD population increased to 809,103 by 2020 (1). While kidney transplantation is the treatment of choice, offering the best prognosis and quality of life for ESRD patients, CRAD remains a leading cause of long-term allograft loss, accounting for nearly 50% of allograft failures within 10 years post-transplantation.

The Banff classification has emphasized interstitial fibrosis and tubular atrophy (IF/TA) as key histopathological features of chronic allograft injury (2). IF/TA represent the common final pathway in various types of CRAD, with fibrosis being a crucial therapeutic target for CRAD. During the fibrotic process, inflammatory infiltration occurs, and graft inflammation is a significant regulatory factor in the progression of fibrosis. Persistent graft inflammation may trigger maladaptive repair responses, leading to excessive matrix accumulation and progressive fibrosis. This ultimately leads to declining graft function and, finally, failure. Exploring the specific mechanism of inflammation in IF/TA is pivotal to improving post-transplantation livability and ESRD morbidity (3).

Interleukin-11 (IL-11), a cytokine of the IL-6 family, has recently attracted attention because of its context-dependent roles in tissue injury and repair. Similar to IL-6, IL-11 initiates signaling by forming superficially similar hexameric complexes with its homologous receptor and gp130. Both cytokines engage in downstream signaling by interacting with their respective α-receptors (IL-6Rα or IL-11Rα) either on the membrane (classical signaling) or in solution (trans-signaling), followed by dimerization of gp130 molecules (4). IL-11RA is expressed in several structural cell populations, including epithelial and stromal cells, and IL-11 signaling has been implicated in diverse fibro-inflammatory disorders, including kidney disease (5). We found that IL-11 can induce sustained activation of ERK in fibroblasts, thereby promoting renal fibrosis (6, 7). Given the high expression of IL-11 in the kidney, we hypothesize that IL-11 also activates the ERK pathway during CRAD and influences inflammatory infiltration in the fibrotic process.

Non-coding RNAs (ncRNAs) are transcripts that generally lack protein-coding capacity but regulate gene expression through multiple mechanisms. A large proportion of the human genome gives rise to non-coding transcripts, including miRNAs, long non-coding RNAs, circular RNAs, and other regulatory RNA species (8). Among these molecules, miRNAs, lncRNAs, and circRNAs have been most frequently investigated in kidney-related pathological processes. For example, miR-142-3p and miR-142-5p have been linked to IF/TA-related pathological changes (9, 10). The lncRNA6524 can act as a sponge for miR-92a-2-5p, thereby enhancing the expression of the Dvl-1/β-catenin signaling pathway and promoting renal fibrosis (11). CircRNA_45478 promotes ischemic acute kidney injury (AKI) by targeting the miR-190a-5p/PHLPP1 axis (12). Among all these ncRNAs, microRNAs (miRNAs), 20-22nt non-coding RNAs, suppress protein synthesis by binding to the 3’-UTR of mRNA, certain miRNAs are capable of concurrently downregulating several genes, thereby directly modulating the expression of biologically related pathways (13). Recently, miRNAs’ role in kidney disease has garnered significant attention. In the past two decades, dozens of miRNAs have been implicated in kidney disease, highlighting their research value in this field. For instance, several initial investigations revealed that the miR-30 family play a crucial role in renal glomerular and tubular damage (14, 15); miR-21 family plays a protective role in the early stage of acute kidney injury, but plays a pro-pathological effect in chronic kidney disease and renal cancer by regulating metabolic, fibrotic and carcinogenic pathways. It is a key molecular target with dual roles (16). Among renal disease-associated miRNAs, miR-204-5p has recently attracted attention because it has been implicated in the protection against diabetic kidney injury, high glucose-induced mesangial cell injury, and renal fibrosis-related pathways. In particular, miR-204-5p overexpression has been shown to ameliorate renal injury and fibrosis through Keap1/Nrf2 signaling, while miR-204-5p-related circRNA networks have been linked to inflammation, oxidative stress, extracellular matrix accumulation, and renal fibrosis (17–19). These findings support the biological plausibility of selecting miR-204-5p as an upstream regulator in CRAD-associated inflammatory and fibrotic injury.

Hence, we aim to elucidate the specific mechanisms by which IL-11 regulates early inflammatory infiltration in CRAD and how miRNA modulate IL-11 expression to regulate CRAD.

## 2 Materials and methods

### 2.1 Cell culture and *in vitro* treatments

BUMPT cells were cultured in high-glucose Dulbecco’s modified Eagle’s medium (DMEM; Gibco, 11965092) supplemented with 10% fetal bovine serum(FBS; Gibco, 10100147) and 1% penicillin-streptomycin at 37°C in a humidified atmosphere containing 5% CO_2_. Cells were passaged when they reached approximately 80-90% confluence.

To establish an *in vitro* model mimicking CRAD-associated early inflammatory and pro-fibrotic responses in renal tubular epithelial cells, BUMPT cells were stimulated with recombinant TGF-β1 at a concentration of 5 ng/mL for 24 h. Control cells were treated with the corresponding vehicle.

To investigate the involvement of IL-11/IL-11RA signaling in TGF-β1-induced inflammatory activation, BUMPT cells were co-treated with TGF-β1 and anti-IL-11 neutralizing antibody (Anti IL-11; R&D Systems, MAB418) or anti-IL-11RA neutralizing antibody (Anti IL-11RA; R&D Systems, AF49) for 24 h. The concentrations of anti-IL-11 and anti-IL-11RA antibodies were both 2 μg/mL. Cells treated with TGF-β1 alone were used as the stimulated control group.

For pathway inhibition experiments, cells were pretreated with PD98059 at 1, 5, or 10 μM for 1 h before stimulation with TGF-β1 or recombinant mouse IL-11 (rmIL-11; Gibco; 220-11-10UG). rmIL-11 was used at a concentration of 5 ng/mL for 24 h. After treatment, cells and culture supernatants were collected for Western blotting, RT-qPCR, and ELISA analyses.

### 2.2 Cell transfection

For siRNA-mediated knockdown experiments, BUMPT cells were seeded into 35-mm culture dishes and cultured until they reached 50-70% confluence. IL-11 siRNA, IL-11RA siRNA was introduced into BUMPT cells using Lipofectamine 2000 (Thermo Fisher, 11668500) following the recommended protocol. The final siRNA concentration was 20 nM. After 6 h of transfection, the medium was replaced with fresh complete medium, and cells were subsequently stimulated with TGF-β1 for 24 h.

For miR-204-5p gain-of-function experiments, BUMPT cells were transfected with miR-204-5p mimic or mimic negative control using the same transfection reagent. The final mimic concentration was 20 nM. Transfection efficiency was confirmed by RT-qPCR. For rescue experiments, rmIL-11 was added to miR-204-5p mimic-transfected BUMPT cells under TGF-β1 stimulation.

### 2.3 Animal model

Female SPF-grade Lewis and F344 rats, aged 6–8 weeks, were purchased from Beijing Vital River Laboratory Animal Technology Co., Ltd. (Beijing, China; license No. SCXK [Jing] 2021-0006; certificate No. 110011241105886583). A total of 50 rats were used in this study. Animals were housed under controlled conditions, including a temperature of 20-26°C, relative humidity of 40-70%, at least 15 air changes per hour, and a 12-h light/dark cycle, with free access to sterile water and SPF-grade maintenance diet. The animal experimental protocol for this project was reviewed and approved by the Animal Welfare and Ethics Committee of Beijing Jinglai Huake Bioscience Technology Co., Ltd. (approval No. JLHK-20240410-01). All animal procedures were performed in the licensed animal facility of Beijing Jinglai Huake Bioscience Technology Co., Ltd. (license No. SYXK [Jing] 2022-0015), which is accredited by the Association for Assessment and Accreditation of Laboratory Animal Care International, and were conducted in accordance with institutional guidelines for laboratory animal welfare.

The chronic renal allograft dysfunction (CRAD) model was established by orthotopic kidney transplantation. F344 rats were used as donors and Lewis rats were used as recipients for allogeneic kidney transplantation. Lewis-to-Lewis kidney transplantation was used as the Sham control. Before surgery, rats were fasted for 12 h with free access to water. All surgical procedures were performed in a clean barrier environment at 24-26°C and 55-65% relative humidity. Rats were anesthetized with isoflurane inhalation using a small-animal anesthesia system. Anesthesia was induced with 4% isoflurane in oxygen at a flow rate of 1.5 L/min for 5 min and then maintained with 2% isoflurane in oxygen at a flow rate of 0.5 L/min throughout the surgical procedure. Adequate anesthesia was confirmed by the absence of pedal withdrawal reflex before surgery, and the physiological status of the rats was continuously monitored throughout the procedure.

For donor surgery, the left kidney was harvested as the donor graft. Rats were heparinized via the lumbar vein with 0.5 mL normal saline containing heparin at 125 U/mL. The abdominal aorta was punctured approximately 1.5 cm below the level of the left renal artery using an indwelling needle. The abdominal aorta was ligated above and below the left renal artery to block renal blood flow, and the left kidney was continuously perfused *in situ* with 0–4°C lactated Ringer’s solution by gravity perfusion from a height of approximately 60 cm. After the kidney became pale yellow and clear perfusate flowed from the renal vein, the left renal artery and vein were transected, and the ureter was dissected and cut at the lower pole of the kidney. The left kidney, together with the ureter, was removed, trimmed, and preserved in heparinized cold saline until transplantation.

For recipient surgery, the native left kidney of Lewis rats was removed after clamping and transecting the left renal artery, renal vein, and ureter. The donor kidney was placed in the left renal fossa. Under a surgical microscope at 10× magnification, the donor renal artery and renal vein were sequentially anastomosed end-to-end to the corresponding recipient vessels using 10–0 atraumatic sutures. Ice chips were used during anastomosis to prevent graft warming. Successful reperfusion was confirmed by the restoration of a bright red and homogeneous appearance of the graft, filling of the renal vein, pulsation of the renal artery, and clear urine outflow from the ureter within 3–5 min. The donor and recipient ureters were then anastomosed intermittently using 10–0 atraumatic sutures. After abdominal closure, 5 mL of warm normal saline was injected intraperitoneally, and penicillin was administered intraperitoneally at 100,000 units. Rats were placed on a heating pad for recovery and continuously monitored after surgery.

To ensure that renal function depended mainly on the transplanted kidney, recipient rats underwent contralateral native nephrectomy on postoperative day 10. Under isoflurane anesthesia, the right kidney was exposed through a flank incision. The right renal artery, renal vein, and ureter were carefully isolated, ligated, and transected, and the right kidney was completely removed. The surgical field was observed for 30–50 s to confirm the absence of bleeding, and the incision was closed layer by layer. Antibiotics were administered for 3 consecutive days after surgery to prevent infection.

The experimental groups included the Sham group (Lewis-to-Lewis kidney transplantation), CRAD group (F344-to-Lewis kidney transplantation), CRAD + PD98059 group, CRAD + IL-11-shRNA AAV group, and CRAD + mmu-miR-204-5p AAV group. For AAV-mediated intervention, IL-11-shRNA AAV or mmu-miR-204-5p overexpression AAV was administered after surgery by tail vein injection at a concentration of 1 × 10^9^ TU/mL, with 100 μL injected per rat. For ERK1/2 inhibition, PD98059 was administered by intraperitoneal injection after surgery at a dose of 0.3 mg/kg. Animals were monitored continuously after treatment.

At 12 weeks post-transplantation, rats were placed in metabolic cages for 24-h urine collection. After urine collection, rats were deeply anesthetized with isoflurane inhalation (4% for induction and 2% for maintenance) using a small-animal anesthesia system, as described above. Animals were subsequently euthanized by continuous inhalation of 4% isoflurane until death. Death was confirmed by the cessation of heartbeat and respiration, together with the absence of pedal withdrawal reflex, before tissue collection. Whole blood and kidney tissues were then immediately harvested. Whole blood samples were centrifuged to obtain serum and stored at −80°C for renal function analysis. Kidney tissues were divided into two parts: one part was fixed in 4% paraformaldehyde for H&E staining, Masson’s trichrome staining, and immunohistochemistry, whereas the remaining tissues were snap-frozen and stored at −80°C for Western blotting, ELISA, and RT-qPCR analyses. Serum creatinine and blood urea nitrogen levels were measured using an automatic biochemical analyzer. Histological and immunohistochemical quantification was performed by investigators blinded to the experimental groups. Animals reaching predefined humane endpoints before the scheduled 12-week endpoint, if any, were euthanized using the same procedure. All animal procedures, including anesthesia, postoperative monitoring, humane endpoint management, and euthanasia, were approved by the Animal Welfare and Ethics Committee of Beijing Jinglai Huake Bioscience Technology Co., Ltd. (Approval No. JLHK-20240410-01) and were conducted in accordance with institutional guidelines for laboratory animal welfare.

### 2.4 Western blotting

Total proteins from kidney tissues or BUMPT cells were extracted using RIPA lysis buffer supplemented with protease and phosphatase inhibitors. Protein concentrations were measured using a NanoPhotometer N50 UV/Vis spectrophotometer (Implen, Germany). Equal amounts of protein 40 μg were mixed with 5× SDS-PAGE loading buffer (Beyotime, P0015L), boiled at 95–100°C for 5 min, separated by SDS-PAGE, and transferred onto PVDF membranes (Millipore, IPVH00010). Protein molecular weight markers (Thermo Fisher, 26616) were used to indicate the molecular weights of target proteins.

After blocking with 5% BSA for 1 h at room temperature, the membranes were incubated with primary antibodies at 4°C overnight. The following primary antibodies were used: IL-11 (Bioss, bs-1827R, 1:1000 dilution), IL-11RA (HUABIO, HA720044, 1:1000 dilution), p-ERK1/2 (Cell Signaling Technology, #9101S, 1:1000 dilution), ERK1/2 (Abcam, ab218017, 1:1000 dilution), p-p65 (Cell Signaling Technology, #3033T, 1:1000 dilution), p65 (Cell Signaling Technology, #8242T, 1:1000 dilution), MCP-1 (Proteintech, 26161-1-AP, 1:1000 dilution), MMP-9 (Proteintech, 27306-1-AP, 1:1000 dilution), ICAM-1 (ABclonal, A24648, 1:1000 dilution), IL-1β (ABclonal, A1112, 1:1000 dilution), and GAPDH (Proteintech, 60004-1-Ig, 1:3000 dilution). The membranes were then washed in TBST and exposed to HRP-labeled secondary antibodies for 1 h at room temperature. Immunoreactive bands were detected with enhanced chemiluminescence and captured using a Tanon 6600 imaging system. Densitometric analysis was performed with ImageJ. The abundance of phosphorylated ERK and p65 was expressed relative to total ERK and p65, respectively, while non-phosphorylated targets were normalized against GAPDH.

### 2.5 Real-time qPCR

RNA was isolated from kidney samples and BUMPT cells with AG RNAex Pro RNA Reagent (Accurate Biology, AG21101) following the supplier’s protocol. Briefly, kidney tissues were homogenized or cultured cells were lysed directly in RNAex Pro reagent. Chloroform was subsequently added to the lysates, followed by vigorous shaking and centrifugation to obtain phase separation. The aqueous phase containing RNA was transferred to a new RNase-free tube, followed by RNA precipitation with isopropanol. After washing with 75% ethanol, the RNA pellet was briefly air-dried and resuspended in RNase-free water. RNA concentration and purity were assessed using a spectrophotometer. Samples with acceptable purity were used for subsequent reverse transcription and quantitative PCR analyses.

For mRNA analysis, reverse transcription was carried out with a commercial cDNA synthesis kit. Quantitative PCR was performed using SYBR Green PCR Master Mix on a real-time PCR system. The primer sequences used for RT-qPCR are listed in **Table 1**. β-Actin and GAPDH were used as the internal control for BUMPT cells and rat mRNA detection respectively. For miR-204-5p detection, reverse transcription was performed using a miRNA-specific reverse transcription kit, and U6 was used as the internal control. Relative expression levels were calculated using the 2*^−ΔΔCt^* method. .

**Table 1:** Primer sequences used for RT-qPCR.

Species	Gene	Forward primer (5′–3′)	Reverse primer (5′–3′)
Rat kidney tissue	GAPDH	GGCAAGTTCAACGGCACAG	CGCCAGTAGACTCCACGACA
Rat kidney tissue	IL-11	TTCCTGGAGTGCTGACAA	GCTAGGCGAGACATCAAG
Rat kidney tissue	MCP-1	CTGCTGCTACTCATTCACT	CTGTCATACTGGTCACTTCTA
Rat kidney tissue	MMP-9	TGACTACGACACAGACAGA	CACCAGCGATAACCATCC
Rat kidney tissue and BUMPT cells/Mouse	U6	CTCGCTTCGGCAGCACA	AACGCTTCACGAATTTGCGT
Rat kidney tissue	miR-204-5p-RT	GTCGTATCCAGTGCAGGGTCCGAGGTATTCGCACTGGATACGACAGGCATAGG	
Rat kidney tissue	miR-204-5p	CGCGTTCCCTTTGTCATCCTGTCG	CAGTGCAGGGTCCGAGGTAT
BUMPT cells/Mouse	β-Actin	GGCTGTATTCCCCTCCATCG	CCAGTTGGTAACAATGCCATGT
BUMPT cells/Mouse	IL-11	ACGCTGGTCATCCCCAAGA	CATCTCGGACAAATTCTGGAGG
BUMPT cells/Mouse	IL-11RA	TAACAGATGCTGTGGCTGGG	GGGGACCAGTGCTAGGAGTA
BUMPT cells/Mouse	MCP-1	TTAAAAACCTGGATCGGAACCAA	GCATTAGCTTCAGATTTACGGGT
BUMPT cells/Mouse	MMP-9	ACGACATAGACGGCATCCA	GCTGTGGTTCAGTTGTGGTG
BUMPT cells/Mouse	miR-204-5p-RT	GTCGTATCCAGTGCAGGGTCCGAGGTATTCGCACTGGATACGACAGGCAT	
BUMPT cells/Mouse	miR-204-5p	CACTGCCTTTCCCTTTGTCATCC	ATCCAGTGCAGGGTCCGAGG

IL-11, interleukin-11; MCP-1, monocyte chemoattractant protein-1; MMP-9, matrix metalloproteinase-9. Rat primers were used for kidney tissue samples, whereas mouse primers were used for BUMPT cells.

### 2.6 Enzyme-linked immunosorbent assay (ELISA)

Protein concentrations of IL-11, MCP-1, MMP-9, and ICAM-1 in kidney tissue homogenates or cell culture supernatants were measured using commercially available ELISA kits according to the manufacturer’s instructions. For kidney tissues, samples were homogenized in ice-cold PBS or lysis buffer, centrifuged at 12000 rpm/min for 20 min at 4°C, and the supernatants were collected. For cell culture supernatants, media were collected and centrifuged to remove debris. Absorbance was measured at 450 nm using a microplate reader. Protein concentrations were calculated based on standard curves.

### 2.7 Histological staining

Kidney tissue was harvested and then embedded in paraffin for various staining procedures as our previous description (20–22). For hematoxylin and eosin (H&E) staining, sections were deparaffinized, rehydrated, stained with hematoxylin and eosin, dehydrated, cleared, and mounted. For Masson’s trichrome staining, sections were stained using a commercial Masson staining kit according to the manufacturer’s instructions.

Images were captured under a light microscope. Fibrotic area was quantified using ImageJ software and expressed as the percentage of Masson-positive area relative to the total tissue area. At least 3 randomly selected non-overlapping fields per sample were analyzed by investigators blinded to group allocation.

### 2.8 Immunohistochemistry

Paraffin-embedded kidney sections were deparaffinized, rehydrated, and subjected to antigen retrieval in citrate buffer (pH 6.0) using microwave heating. Endogenous peroxidase activity was blocked with 3% hydrogen peroxide for 10 min, followed by blocking with 5% bovine serum albumin for 30 min at room temperature. Sections were incubated overnight at 4°C with primary antibodies against IL-11(1:100 dilution), p-ERK1/2(1:100 dilution), p-p65(1:100 dilution), and CD68(1:100 dilution). After washing with PBS, sections were incubated with HRP-conjugated secondary antibodies at room temperature. Immunoreactivity was visualized using DAB substrate, and nuclei were counterstained with hematoxylin.

Images were captured under a light microscope. The positive staining area was quantified using ImageJ software and expressed as the percentage of DAB-positive area relative to the total tissue area. At least 3 randomly selected fields per section were analyzed by blinded investigators.

### 2.9 Dual-luciferase reporter gene assay

The wild-type fragment of the IL-11 3′-UTR containing the predicted miR-204-5p binding site and the corresponding mutant sequence were cloned into a luciferase reporter vector. Cells were co-transfected with IL-11 3′-UTR-WT or IL-11 3′-UTR-Mut reporter plasmids together with miR-204-5p mimic or mimic negative control using Lipofectamine 2000. After 24 h, luciferase activity was measured using a dual-luciferase reporter assay system according to the manufacturer’s instructions. Firefly luciferase activity was normalized to Renilla luciferase activity.

### 2.10 Immunofluorescence staining

BUMPT cells were grown to 30-40% confluency in 12-well plates plated with cell slides, then slides were washed three times with 4°C PBS, fixed with 4% paraformaldehyde for 10 min at 4°C, permeabilized with 0.2% Triton X-100 and blocked with 5% bovine serum albumin (BSA) for 1 hour at room temperature. Next, slides were incubated with IL-11RA antibody (1:50 dilution) overnight at 4°C. Cells were subsequently washed 3 times with 1% BSA and incubated with secondary antibody for 1 hour at room temperature. At last, the fixed cells were stained with DAPI at room temperature for 15 min, and a fluorescence microscope was performed to capture images.

### 2.11 Renal function analysis

Serum creatinine and blood urea nitrogen levels were measured using an automatic biochemical analyzer or commercial assay kits according to the manufacturer’s instructions. These parameters were used to evaluate renal function in each experimental group.

### 2.12 Prediction of miRNAs targeting IL-11 mRNA

To predict upstream targets of IL-11 mRNA, we used a combination of computational approaches utilizing established databases and bioinformatics tools. To identify upstream target genes, we utilized three widely recognized databases: miRDB (https://mirdb.org/), miRwalk (http://mirwalk.umm.uni-heidelberg.de/), and TargetScan (https://www.targetscan.org/). After extracting target gene lists from each of these databases, we identified the overlapping genes—those predicted targets that appeared in all three databases—for further experimental validation through relevant assays, including Dual-luciferase reporter gene assay and RT-qPCR, to confirm their regulation by miRNA and their involvement in the functional pathways related to our research focus.

### 2.13 Image quantification and analysis

Histological and immunohistochemical images were analyzed using ImageJ software. For Masson’s trichrome staining, fibrotic area was calculated as the percentage of collagen-positive area relative to the total tissue area. For immunohistochemistry, positive staining area was calculated as the percentage of DAB-positive area relative to the total tissue area. For Western blotting, band intensities were quantified using ImageJ. Quantification was performed using identical threshold settings within the same experiment. All image analyses were performed by investigators blinded to group allocation.

### 2.14 Statistics

Data are presented as mean ± standard deviation (SD). For animal experiments, n represents the number of rats in each group. For *in vitro* experiments, data were obtained from at least six independent experiments. Statistical analyses were performed using GraphPad Prism 10. Comparisons among multiple groups were performed using one-way analysis of variance followed by Tukey’s *post hoc* test. A value of P < 0.05 was considered statistically significant.

## 3 Results

### 3.1 IL-11 inhibition attenuates renal inflammatory infiltration and injury in early-stage CRAD

**Figure 1:**
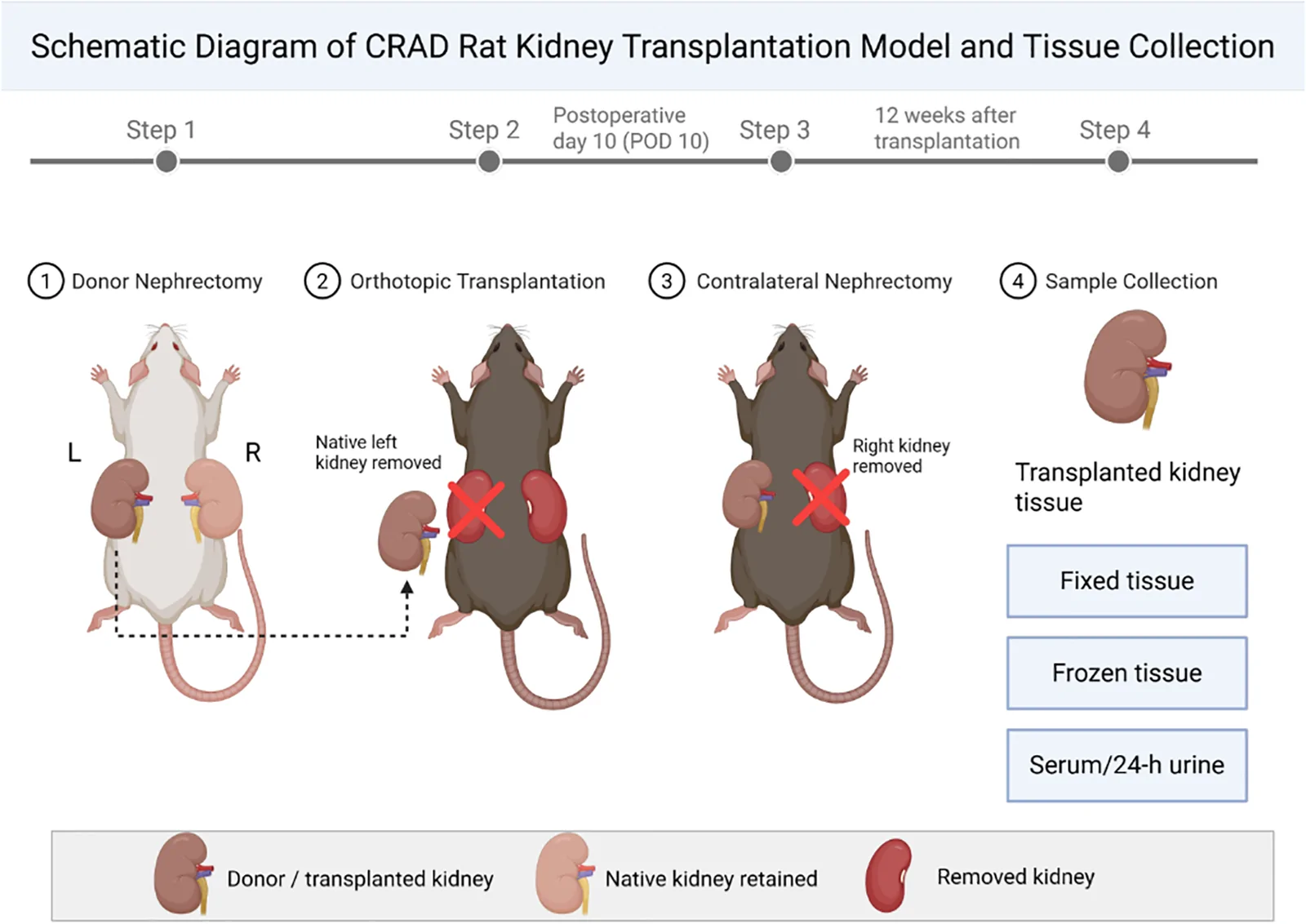
Schematic workflow of early-stage CRAD model establishment and sample collection. Schematic illustration of the experimental workflow for establishing the chronic renal allograft dysfunction (CRAD) rat model. Orthotopic kidney transplantation was performed, followed by contralateral nephrectomy on postoperative day 10. At 12 weeks post-transplantation, 24-h urine samples were collected using metabolic cages, and serum and kidney tissues were harvested for subsequent histological, biochemical, and molecular analyses. The 12-week time point was used to assess early inflammatory and fibrotic changes during CRAD progression. Created with BioRender.com under a publication license.

To establish an early-stage CRAD model, orthotopic kidney transplantation was performed in rats, followed by contralateral nephrectomy on postoperative day 10. Kidney tissues, serum, and 24-h urine samples were collected at 12 weeks post-transplantation to evaluate early inflammatory and fibrotic changes during CRAD progression (**Figure 1**).

**Figure 2:**
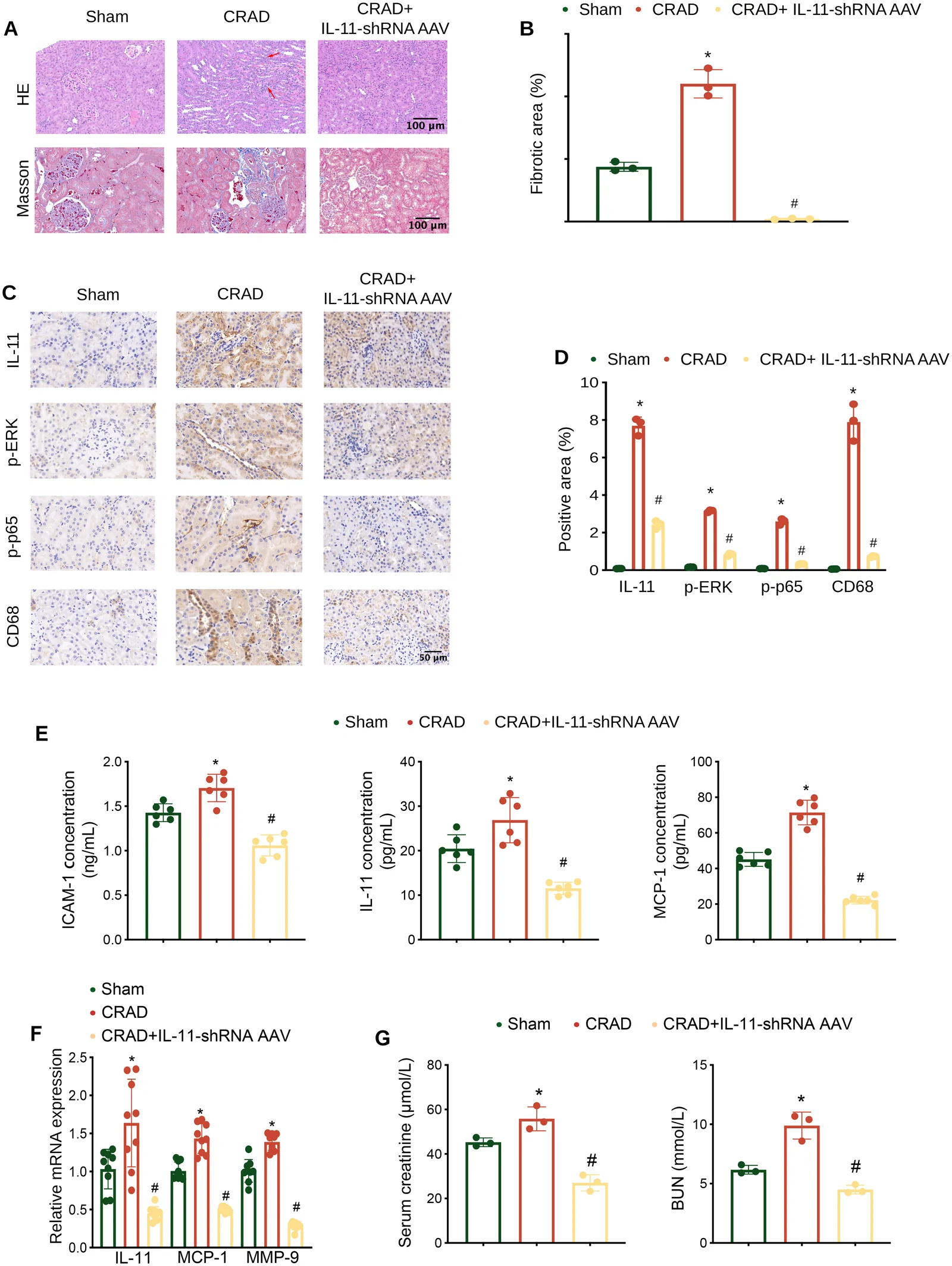
IL-11 inhibition attenuates renal inflammatory infiltration and injury in early-stage CRAD. **(A)** Representative H&E and Masson’s trichrome staining images of kidney tissues from the Sham, CRAD, and CRAD + IL-11-shRNA AAV groups. Red arrows indicate representative early CRAD-associated tubulointerstitial injury, including inflammatory cell infiltration, interstitial expansion, and tubular structural disorganization. Scale bar, 100 μm. **(B)** Quantitative analysis of fibrotic area based on Masson’s trichrome staining. **(C)** Representative immunohistochemical staining images of IL-11, p-ERK, p-p65, and CD68 in kidney tissues. Scale bars, 50 μm. **(D)** Quantitative analysis of IL-11-, p-ERK-, p-p65-, and CD68-positive areas. **(E)** Protein concentrations of ICAM-1, IL-11, and MCP-1 in kidney tissues. **(F)** Relative mRNA expression levels of IL-11, MCP-1, and MMP-9 in kidney tissues. **(G)** Serum creatinine and blood urea nitrogen (BUN) levels in each group. Data are presented as mean ± SD; n = 3 rats per group. ^*^P < 0.05 vs. Sham group; ^#^*P* < 0.05 vs. CRAD group.

Compared with the Sham group, CRAD rats exhibited evident renal histopathological injury. H&E staining showed tubular-interstitial structural damage and inflammatory cell infiltration, whereas Masson’s trichrome staining revealed increased collagen deposition and fibrotic remodeling in CRAD kidneys (**Figure 2A**). Quantitative analysis confirmed that the fibrotic area was significantly increased in the CRAD group compared with the Sham group (**Figure 2B**).

To determine whether IL-11 contributes to CRAD-associated renal injury, IL-11 expression was inhibited *in vivo* using IL-11-shRNA AAV. Immunohistochemical staining showed that IL-11 expression was markedly increased in CRAD kidneys and was reduced after IL-11-shRNA AAV treatment (**Figures 2C, D**). In parallel, CRAD kidneys showed increased p-ERK, p-p65, and CD68-positive macrophage accumulation, whereas IL-11 knockdown reduced p-ERK and p-p65 expression and attenuated CD68-positive inflammatory cell infiltration (**Figures 2C, D**).

Consistent with these histological changes, IL-11-shRNA AAV treatment improved renal function, as shown by decreased serum creatinine and blood urea nitrogen levels compared with the CRAD group (**Figure 2E**). RT-qPCR analysis further showed that IL-11 knockdown reduced the mRNA expression levels of IL-11, MCP-1, and MMP-9 in CRAD kidneys (**Figure 2F**). Similarly, protein levels of ICAM-1, IL-11, and MCP-1 were elevated in CRAD kidneys and decreased after IL-11-shRNA AAV treatment (**Figure 2G**).

Collectively, these findings demonstrate that early-stage CRAD is characterized by renal inflammatory infiltration, fibrotic remodeling, IL-11 upregulation, and activation of ERK1/2-NF-κB-associated inflammatory signaling. Inhibition of IL-11 attenuates renal inflammatory injury and functional impairment in early-stage CRAD.

### 3.2 IL-11/IL-11RA signaling contributes to inflammatory activation in TGF-β1-stimulated BUMPT cells

**Figure 3:**
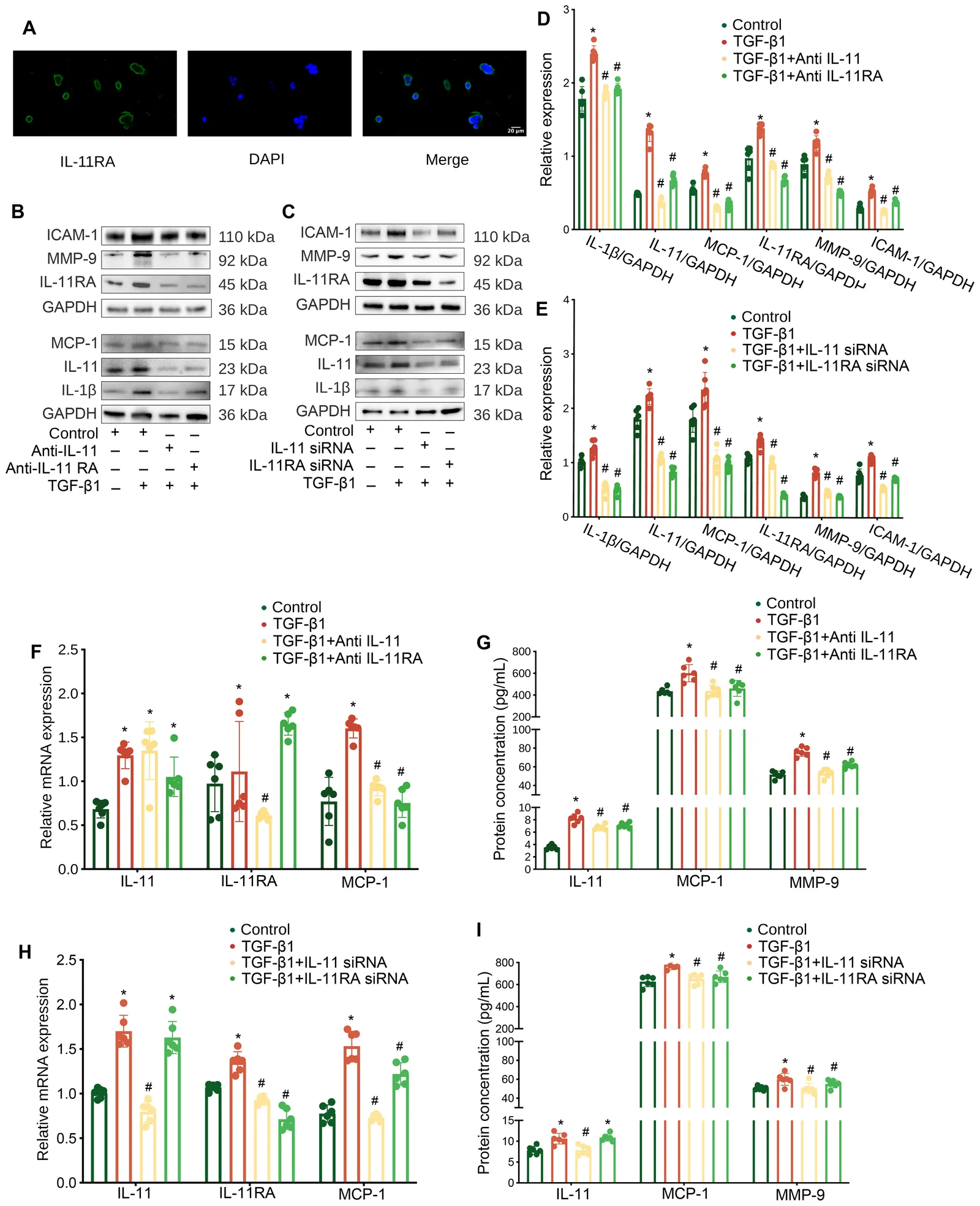
IL-11/IL-11RA signaling contributes to inflammatory activation in TGF-β1-stimulated BUMPT cells. **(A)** Representative immunofluorescence staining images showing IL-11RA expression in BUMPT cells. Nuclei were stained with DAPI. Scale bar, 20 μm. **(B)** Representative immunoblotting images showing the effects of anti-IL-11 and anti-IL-11RA antibodies on the expression of ICAM-1, MMP-9, IL-11RA, MCP-1, IL-11, and IL-1β in TGF-β1-stimulated BUMPT cells. **(C)** Representative immunoblotting images showing the effects of IL-11 siRNA and IL-11RA siRNA on inflammatory mediator expression in TGF-β1-stimulated BUMPT cells. **(D)** Quantitative analysis of protein expression in panel **(B)**. **(E)** Quantitative analysis of protein expression in panel **(C)**. **(F)** Relative mRNA expression levels of IL-11, IL-11RA, and MCP-1 after anti-IL-11 or anti-IL-11RA treatment. **(G)** Protein concentrations of IL-11, MCP-1, and MMP-9 in culture supernatants after anti-IL-11 or anti-IL-11RA treatment. **(H)** Relative mRNA expression levels of IL-11, IL-11RA, and MCP-1 after IL-11 or IL-11RA knockdown. **(I)** Protein concentrations of IL-11, MCP-1, and MMP-9 in culture supernatants after IL-11 or IL-11RA knockdown. Data are presented as mean ± SD; n = 3 independent experiments. ^*^P < 0.05 vs. Control group; ^#^*P* < 0.05 vs. TGF-β1 group.

To further investigate whether IL-11-associated signaling contributes to tubular epithelial inflammatory activation, BUMPT cells were stimulated with TGF-β1 to establish an *in vitro* model mimicking CRAD-associated early inflammatory and pro-fibrotic responses. Immunofluorescence staining confirmed the presence of IL-11RA in BUMPT cells, suggesting that these cells are capable of responding to IL-11-related signaling (**Figure 3A**).

TGF-β1 stimulation markedly increased the protein expression of IL-11, IL-11RA, and inflammatory mediators, including MCP-1, MMP-9, ICAM-1, and IL-1β (**Figures 3B–E**). To determine whether IL-11 and IL-11RA were functionally involved in this process, BUMPT cells were treated with anti-IL-11 or anti-IL-11RA antibodies under TGF-β1 stimulation. Neutralization of IL-11 or IL-11RA attenuated the TGF-β1-induced upregulation of inflammatory mediators at the protein level (**Figures 3B, D**). Consistently, blockade of IL-11 or IL-11RA reduced the mRNA expression of IL-11, IL-11RA, and MCP-1 to varying degrees (**Figure 3F**). ELISA analysis further showed that anti-IL-11 or anti-IL-11RA treatment decreased the secretion of IL-11, MCP-1, and MMP-9 in TGF-β1-stimulated BUMPT cells (**Figure 3G**).

We further performed siRNA-mediated knockdown of IL-11 or IL-11RA. Knockdown of IL-11 reduced IL-11 expression and suppressed the TGF-β1-induced expression of inflammatory mediators, whereas IL-11RA knockdown also attenuated the expression of MCP-1, MMP-9, ICAM-1, and IL-1β (**Figures 3C, E**). In line with these protein-level changes, IL-11 or IL-11RA knockdown decreased the mRNA expression of inflammatory-related genes under TGF-β1 stimulation (**Figure 3H**). ELISA results further confirmed that silencing IL-11 or IL-11RA reduced the secretion of MCP-1 and MMP-9 in TGF-β1-stimulated BUMPT cells (**Figure 3I**).

Collectively, these findings suggest that IL-11/IL-11RA signaling contributes to the inflammatory activation of renal tubular epithelial cells under TGF-β1-induced early inflammatory and pro-fibrotic conditions.

### 3.3 ERK1/2 inhibition attenuates NF-κB activation and inflammatory injury in early-stage CRAD

**Figure 4:**
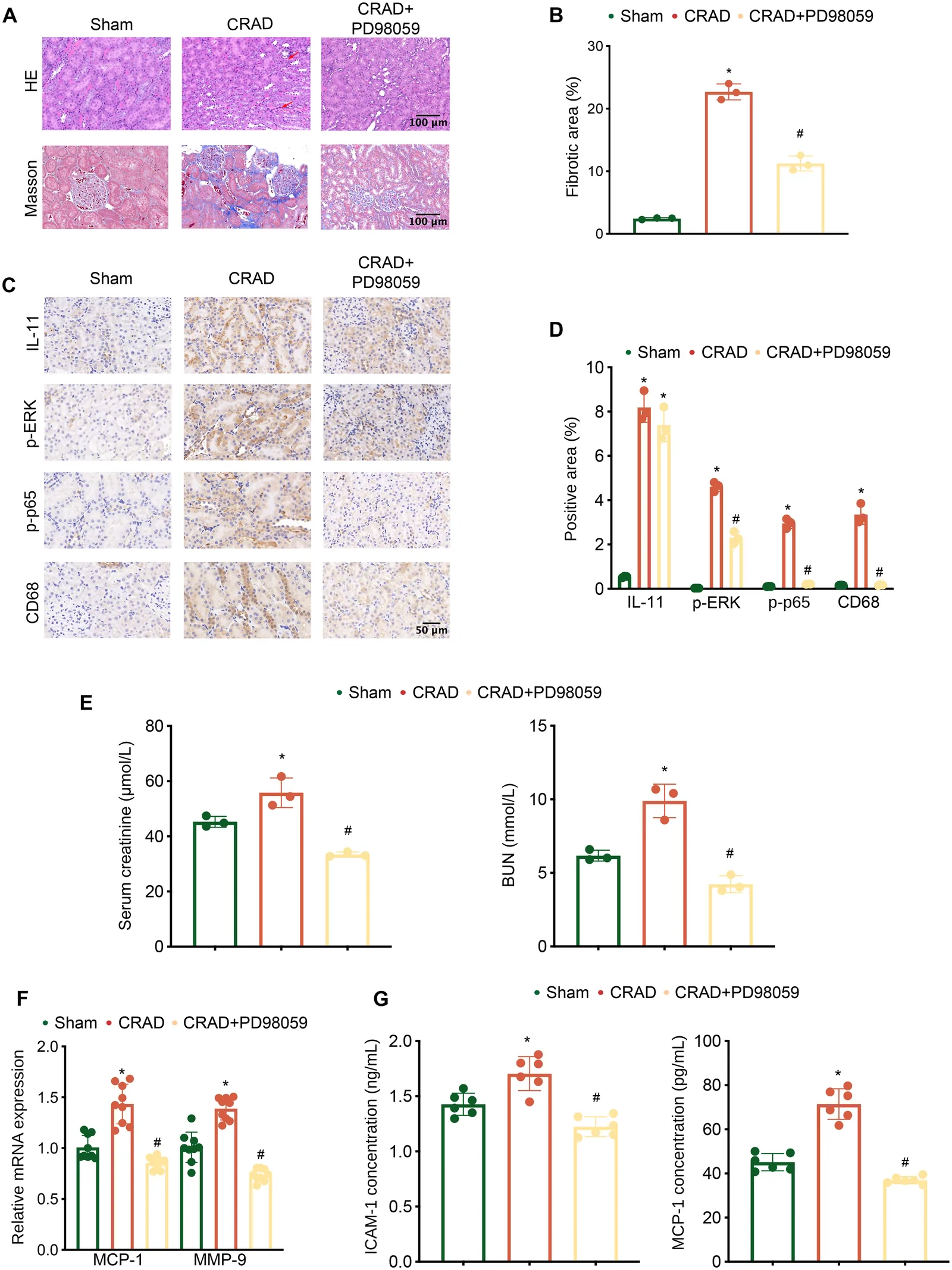
ERK1/2 inhibition attenuates NF-κB activation and inflammatory injury in early-stage CRAD. **(A)** Representative H&E and Masson’s trichrome staining images of kidney tissues from the Sham, CRAD, and CRAD+PD98059 groups. Red arrows indicate representative areas of inflammatory cell infiltration and tubulointerstitial structural disruption in CRAD kidneys. Scale bars, 100 μm. **(B)** Quantitative analysis of fibrotic area based on Masson’s trichrome staining. **(C)** Representative immunohistochemical staining images of IL-11, p-ERK, p-p65, and CD68 in kidney tissues. Scale bars, 50 μm. **(D)** Quantitative analysis of IL-11-, p-ERK-, p-p65-, and CD68-positive areas. **(E)** Serum creatinine and BUN levels in each group. **(F)** Relative mRNA expression levels of MCP-1 and MMP-9 in kidney tissues. **(G)** Protein concentrations of ICAM-1 and MCP-1 in kidney tissues. Data are presented as mean ± SD; n = 3 rats per group. ^*^P < 0.05 vs. Sham group; ^#^*P* < 0.05 vs. CRAD group.

To determine whether ERK1/2 signaling participates in CRAD-associated inflammatory injury, CRAD rats were treated with PD98059, a pharmacological inhibitor of ERK1/2 activation. Histological analysis showed that CRAD kidneys exhibited obvious tubular-interstitial injury, inflammatory cell infiltration, and collagen deposition compared with the Sham group. PD98059 treatment alleviated these pathological changes, as shown by H&E and Masson’s trichrome staining (**Figure 4A**). Quantitative analysis further confirmed that the fibrotic area was significantly increased in CRAD kidneys and was reduced after PD98059 treatment (**Figure 4B**).

We next examined the activation of IL-11-associated downstream inflammatory signaling in kidney tissues. Immunohistochemical staining showed that IL-11, p-ERK, p-p65, and CD68 were markedly increased in CRAD kidneys compared with Sham controls (**Figures 4C, D**). After PD98059 treatment, p-ERK and p-p65 expression were significantly reduced, accompanied by decreased CD68-positive macrophage accumulation (**Figures 4C, D**). In contrast, IL-11 expression was only modestly affected by PD98059 treatment, suggesting that IL-11 may act upstream of ERK1/2 activation in CRAD-associated inflammatory signaling.

Consistent with the improvement in histological injury, PD98059 treatment significantly reduced serum creatinine and blood urea nitrogen levels in CRAD rats, indicating improved renal function (**Figure 4E**). Moreover, RT-qPCR analysis showed that the mRNA expression levels of MCP-1 and MMP-9 were markedly elevated in CRAD kidneys and were significantly decreased after PD98059 treatment (**Figure 4F**). Similarly, the protein levels of ICAM-1 and MCP-1 were increased in CRAD kidneys, whereas PD98059 treatment attenuated their upregulation (**Figure 4G**).

Collectively, these findings indicate that ERK1/2 inhibition suppresses NF-κB activation, reduces inflammatory mediator expression, and attenuates renal inflammatory injury in early-stage CRAD. These results further support the involvement of the ERK1/2-NF-κB signaling axis in CRAD-associated inflammatory responses.

### 3.4 IL-11 promotes inflammatory activation through ERK1/2-NF-κB-associated signaling in BUMPT cells

**Figure 5:**
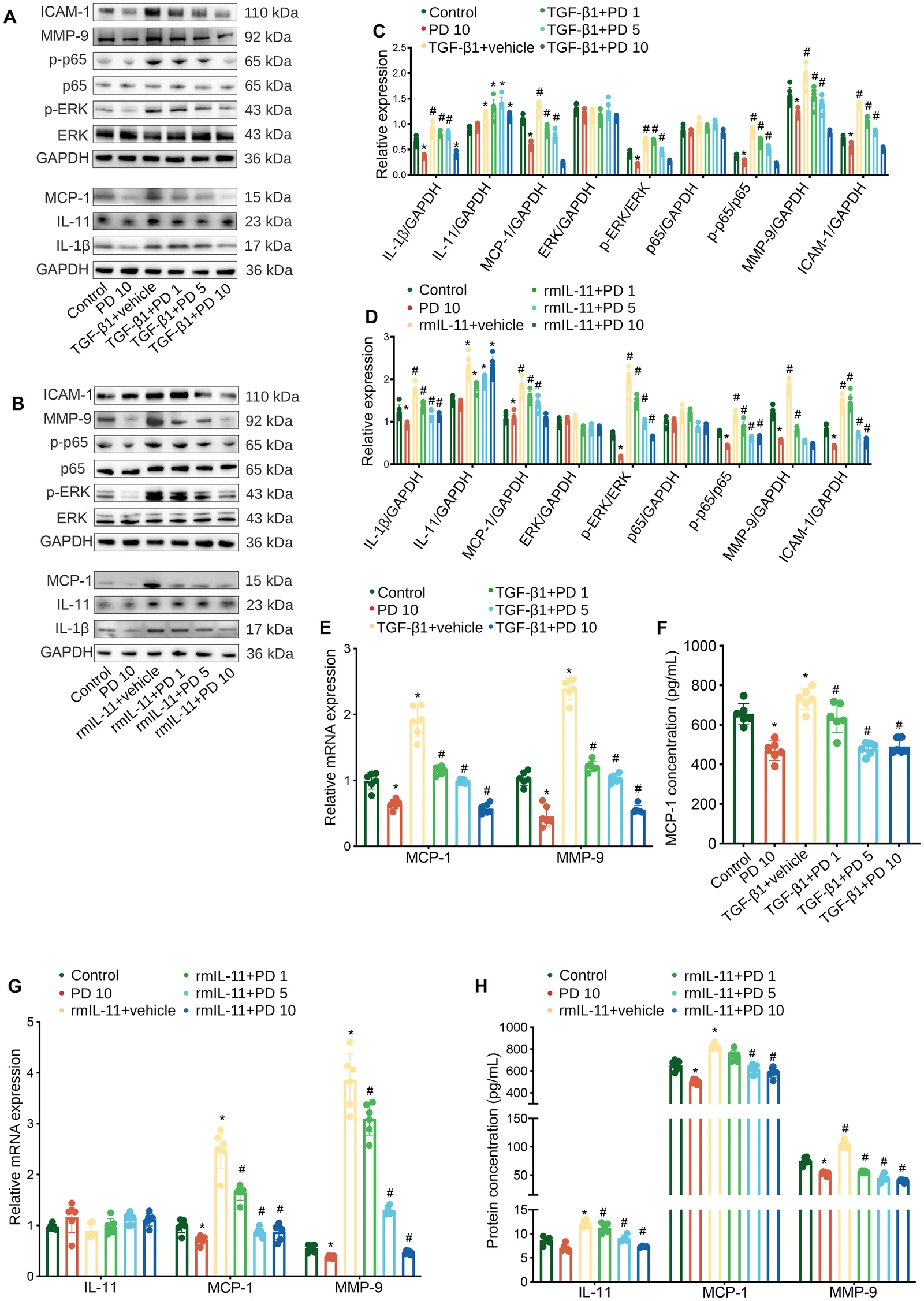
PD98059 suppresses TGF-β1- and rmIL-11-induced ERK1/2-NF-κB activation and inflammatory mediator expression in BUMPT cells. **(A)** Representative immunoblotting images showing the effects of different concentrations of PD98059 on TGF-β1-induced expression of ICAM-1, MMP-9, p-p65, p65, p-ERK, ERK, MCP-1, IL-11, and IL-1β in BUMPT cells. **(B)** Representative immunoblotting images showing the effects of different concentrations of PD98059 on rmIL-11-induced ERK1/2-NF-κB activation and inflammatory mediator expression in BUMPT cells. **(C)** Quantitative analysis of protein expression in panel **(A)**. **(D)** Quantitative analysis of protein expression in panel **(B)**. **(E)** Relative mRNA expression levels of MCP-1 and MMP-9 in TGF-β1-stimulated BUMPT cells treated with PD98059. **(F)** MCP-1 concentration in culture supernatants from TGF-β1-stimulated BUMPT cells treated with PD98059. **(G)** Relative mRNA expression levels of IL-11, MCP-1, and MMP-9 in rmIL-11-stimulated BUMPT cells treated with PD98059. **(H)** Protein concentrations of IL-11, MCP-1, and MMP-9 in culture supernatants from rmIL-11-stimulated BUMPT cells treated with PD98059. Data are presented as mean ± SD; n = 6 independent experiments. PD 1, PD 5, and PD 10 indicate PD98059 at 1, 5, and 10 μM, respectively. ^*^P < 0.05 vs. Control group; ^#^*P* < 0.05 vs. TGF-β1 + vehicle or rmIL-11 + vehicle group.

To further clarify whether IL-11 activates inflammatory signaling through the ERK1/2-NF-κB axis in renal tubular epithelial cells, BUMPT cells were treated with TGF-β1 or rmIL-11 in the presence or absence of different concentrations of PD98059. PD98059 alone did not induce obvious inflammatory activation compared with the Control group, whereas TGF-β1 stimulation markedly increased the expression of IL-11, p-ERK, p-p65, and inflammatory mediators, including MCP-1, MMP-9, ICAM-1, and IL-1β (**Figures 5A, C**).

After PD98059 treatment, TGF-β1-induced ERK1/2 activation was significantly inhibited, as indicated by reduced p-ERK/ERK levels. In parallel, NF-κB activation was attenuated, as reflected by decreased p-p65/p65 expression. The protein expression of MCP-1, MMP-9, ICAM-1, and IL-1β was also reduced following PD98059 treatment (**Figures 5A, C**). Consistently, RT-qPCR analysis showed that PD98059 decreased the mRNA expression of MCP-1 and MMP-9 in TGF-β1-stimulated BUMPT cells (**Figure 5E**). ELISA analysis further confirmed that MCP-1 secretion was reduced after ERK1/2 inhibition (**Figure 5F**).

To determine whether IL-11 directly contributes to this inflammatory signaling cascade, BUMPT cells were stimulated with rmIL-11. rmIL-11 treatment increased p-ERK/ERK and p-p65/p65 levels and upregulated inflammatory mediators, including MCP-1, MMP-9, ICAM-1, and IL-1β (**Figures 5B, D**). Importantly, PD98059 treatment suppressed rmIL-11-induced ERK1/2 and NF-κB activation and reduced the expression of downstream inflammatory mediators (**Figures 5B, D**). In agreement with these protein-level changes, rmIL-11 increased the mRNA expression of MCP-1 and MMP-9, whereas PD98059 attenuated this upregulation (**Figure 5G**). ELISA results further showed that PD98059 reduced rmIL-11-induced secretion of MCP-1 and MMP-9 (**Figure 5H**).

Collectively, these findings suggest that IL-11 promotes inflammatory activation in BUMPT cells through ERK1/2-NF-κB-associated signaling, and that inhibition of ERK1/2 attenuates both TGF-β1- and rmIL-11-induced inflammatory mediator expression.

### 3.5 miR-204-5p directly targets IL-11 and attenuates inflammatory mediator expression in TGF-β1-stimulated BUMPT cells

**Figure 6:**
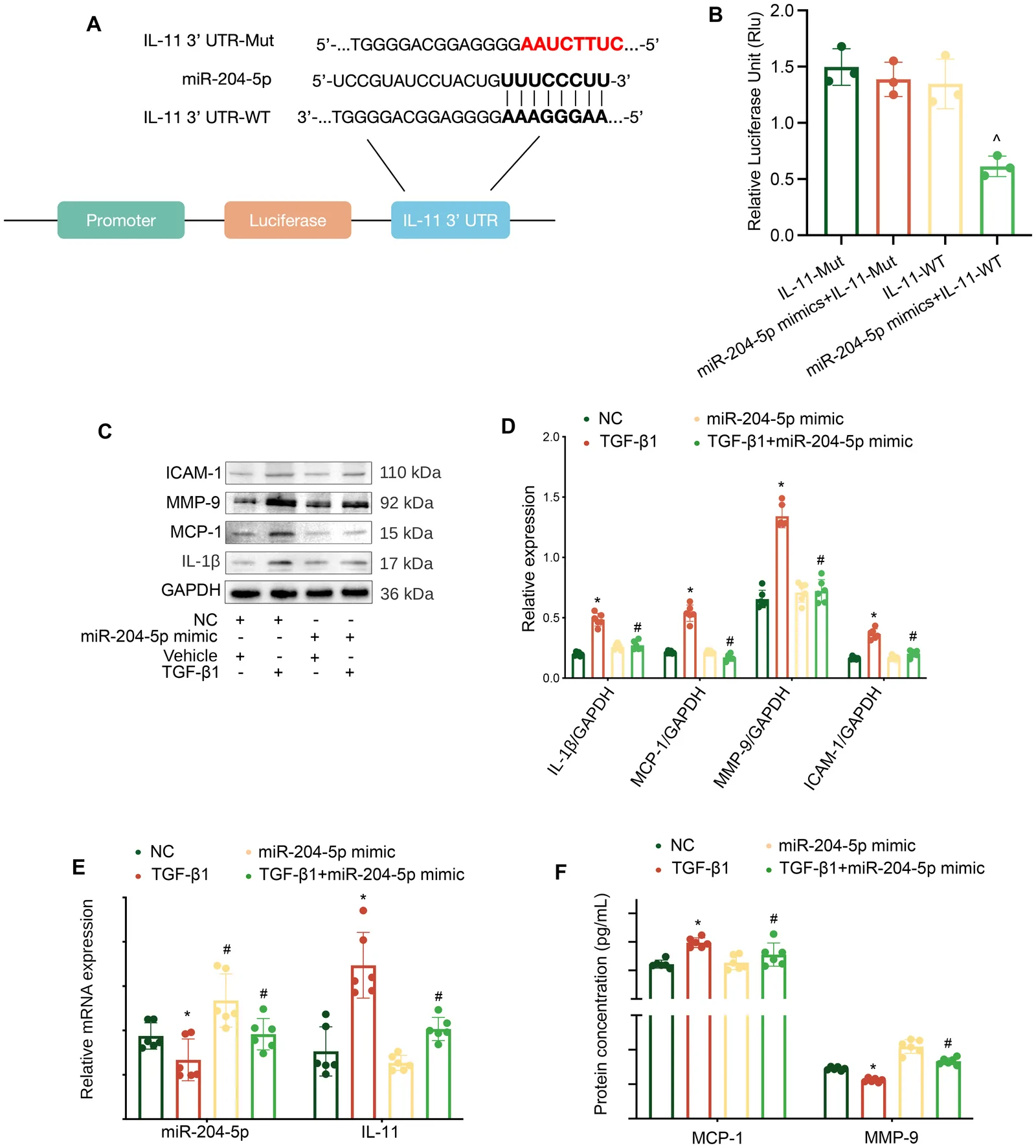
miR-204-5p directly targets IL-11 and attenuates inflammatory mediator expression in TGF-β1-stimulated BUMPT cells. **(A)** Schematic illustration of the predicted miR-204-5p binding site within the IL-11 3′-UTR and the corresponding wild-type and mutant luciferase reporter constructs. **(B)** Dual-luciferase reporter assay showing the effect of miR-204-5p mimic on luciferase activity in cells transfected with IL-11 3′-UTR wild-type or mutant reporter constructs. **(C)** Representative immunoblotting images showing the effects of miR-204-5p mimic on ICAM-1, MMP-9, MCP-1, and IL-1β expression in TGF-β1-stimulated BUMPT cells. **(D)** Quantitative analysis of protein expression in panel **(C)**. **(E)** Relative RNA expression levels of miR-204-5p and IL-11 in BUMPT cells. **(F)** Protein concentrations of MCP-1 and MMP-9 in culture supernatants. The broken y-axis indicates different concentration ranges. Data are presented as mean ± SD; n = 6 independent experiments. ^*^P < 0.05 vs. NC/Control group; ^#^*P* < 0.05 vs. TGF-β1 group; ^^^*P* < 0.05 vs. IL-11 3′-UTR-WT reporter group. WT, wild-type; Mut, mutant; NC, negative control.

To identify potential upstream regulators of IL-11, bioinformatic prediction was used to screen miRNAs with putative binding sites in the IL-11 3′-UTR. Among the candidates, miR-204-5p was selected for further validation because of its predicted interaction with IL-11 (**Figure 6A**). Dual-luciferase reporter assays showed that miR-204-5p mimic reduced the activity of the wild-type IL-11 3′-UTR reporter. This inhibitory effect was lost when the predicted binding sequence was mutated (**Figure 6B**), indicating a direct interaction between miR-204-5p and the IL-11 3′-UTR.

We then assessed whether miR-204-5p regulates IL-11 expression in BUMPT cells. TGF-β1 reduced miR-204-5p expression and increased IL-11 mRNA levels. Transfection with miR-204-5p mimic restored miR-204-5p abundance and decreased IL-11 expression under TGF-β1 stimulation (**Figure 6E**). These data suggest a negative regulatory relationship between miR-204-5p and IL-11.

At the functional level, miR-204-5p mimic decreased the TGF-β1-induced expression of MCP-1, MMP-9, ICAM-1, and IL-1β (**Figures 6C, D**). In culture supernatants, miR-204-5p mimic also reduced inflammatory mediator secretion, as shown by ELISA (**Figure 6F**).

Thus, miR-204-5p directly binds the IL-11 3′-UTR and suppresses IL-11 expression, thereby attenuating TGF-β1-induced inflammatory activation in BUMPT cells.

### 3.6 rmIL-11 rescues the inhibitory effects of miR-204-5p mimic on inflammatory signaling, and miR-204-5p overexpression attenuates renal inflammatory injury *in vivo*

**Figure 7:**
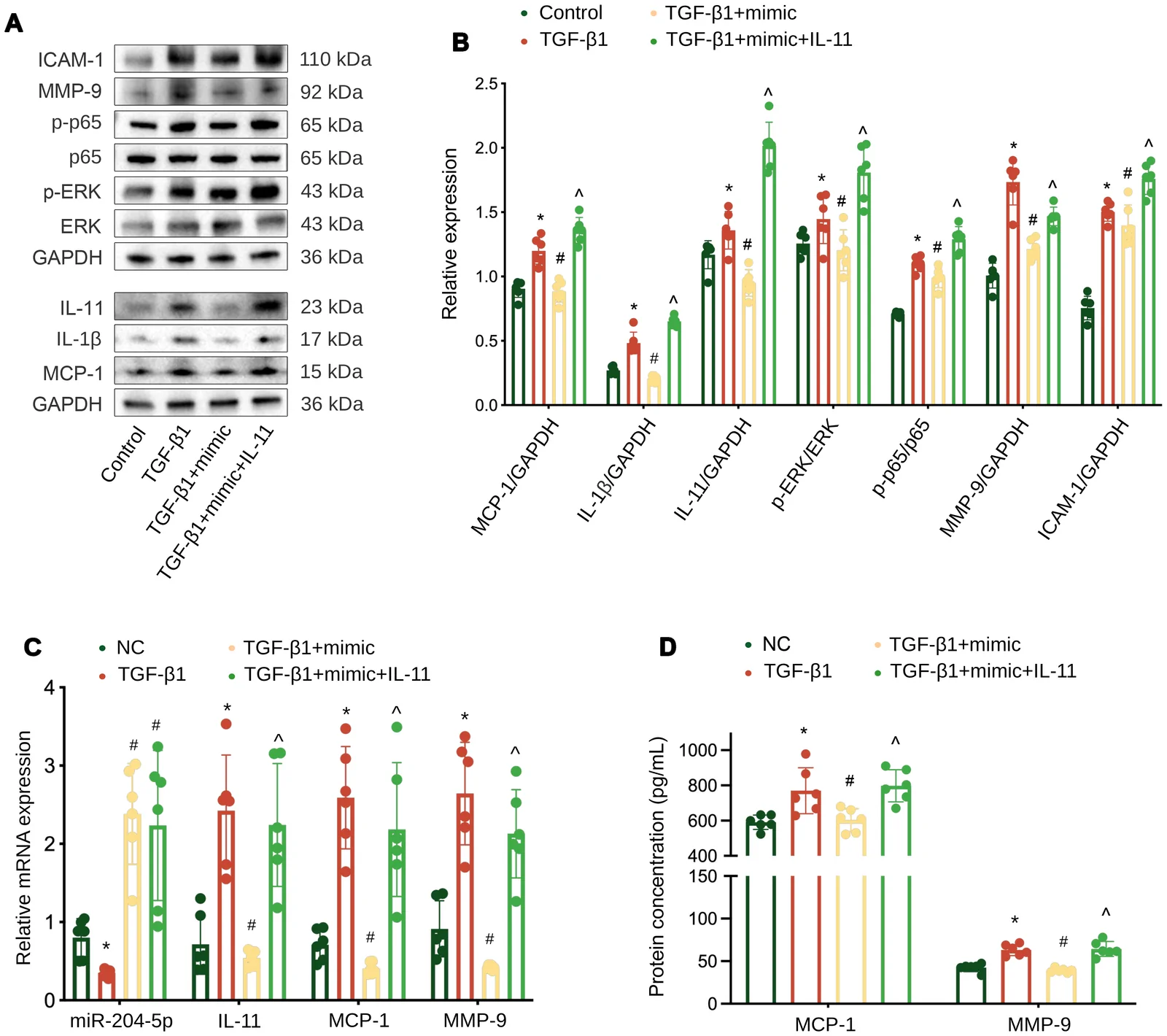
rmIL-11 partially rescues the inhibitory effects of miR-204-5p mimic on ERK1/2-NF-κB inflammatory signaling in BUMPT cells. **(A)** Representative immunoblotting images showing the effects of rmIL-11 rescue on ERK1/2-NF-κB activation and inflammatory mediator expression in miR-204-5p mimic-transfected BUMPT cells under TGF-β1 stimulation. **(B)** Quantitative analysis of protein expression in panel A, including p-ERK/ERK, p-p65/p65, MCP-1/GAPDH, MMP-9/GAPDH, ICAM-1/GAPDH, and IL-1β/GAPDH. **(C)** Relative RNA expression levels of miR-204-5p, IL-11, MCP-1, and MMP-9 in rescue experiments. **(D)** Protein concentrations of MCP-1 and MMP-9 in culture supernatants. The broken y-axis indicates different concentration ranges. Data are presented as mean ± SD; n = 6 independent experiments. ^*^P < 0.05 vs. Control group; ^#^*P* < 0.05 vs. TGF-β1 group; ^^^*P* < 0.05 vs. TGF-β1 + miR-204-5p mimic group.

To further determine whether IL-11 is a functional downstream mediator of miR-204-5p, rescue experiments were performed by adding rmIL-11 to miR-204-5p mimic-transfected BUMPT cells under TGF-β1 stimulation. As expected, miR-204-5p mimic reduced TGF-β1-induced ERK1/2 and NF-κB activation, as indicated by decreased p-ERK/ERK and p-p65/p65 levels. The expression of downstream inflammatory mediators, including MCP-1, MMP-9, ICAM-1, and IL-1β, was also suppressed by miR-204-5p mimic (**Figures 7A, B**).

Notably, rmIL-11 treatment partially restored ERK1/2 and NF-κB activation in miR-204-5p mimic-transfected cells. Consistently, rmIL-11 partially reversed the inhibitory effects of miR-204-5p mimic on inflammatory mediator expression at the protein level (**Figures 7A, B**). RT-qPCR analysis further showed that miR-204-5p mimic decreased IL-11, MCP-1, and MMP-9 RNA expression, whereas rmIL-11 treatment partially restored the expression of MCP-1 and MMP-9 (**Figure 7C**). ELISA analysis also showed that miR-204-5p mimic reduced MCP-1 and MMP-9 secretion, whereas rmIL-11 partially restored their secretion levels (**Figure 7D**). These findings support IL-11 as a functional downstream mediator of miR-204-5p in TGF-β1-stimulated BUMPT cells.

**Figure 8:**
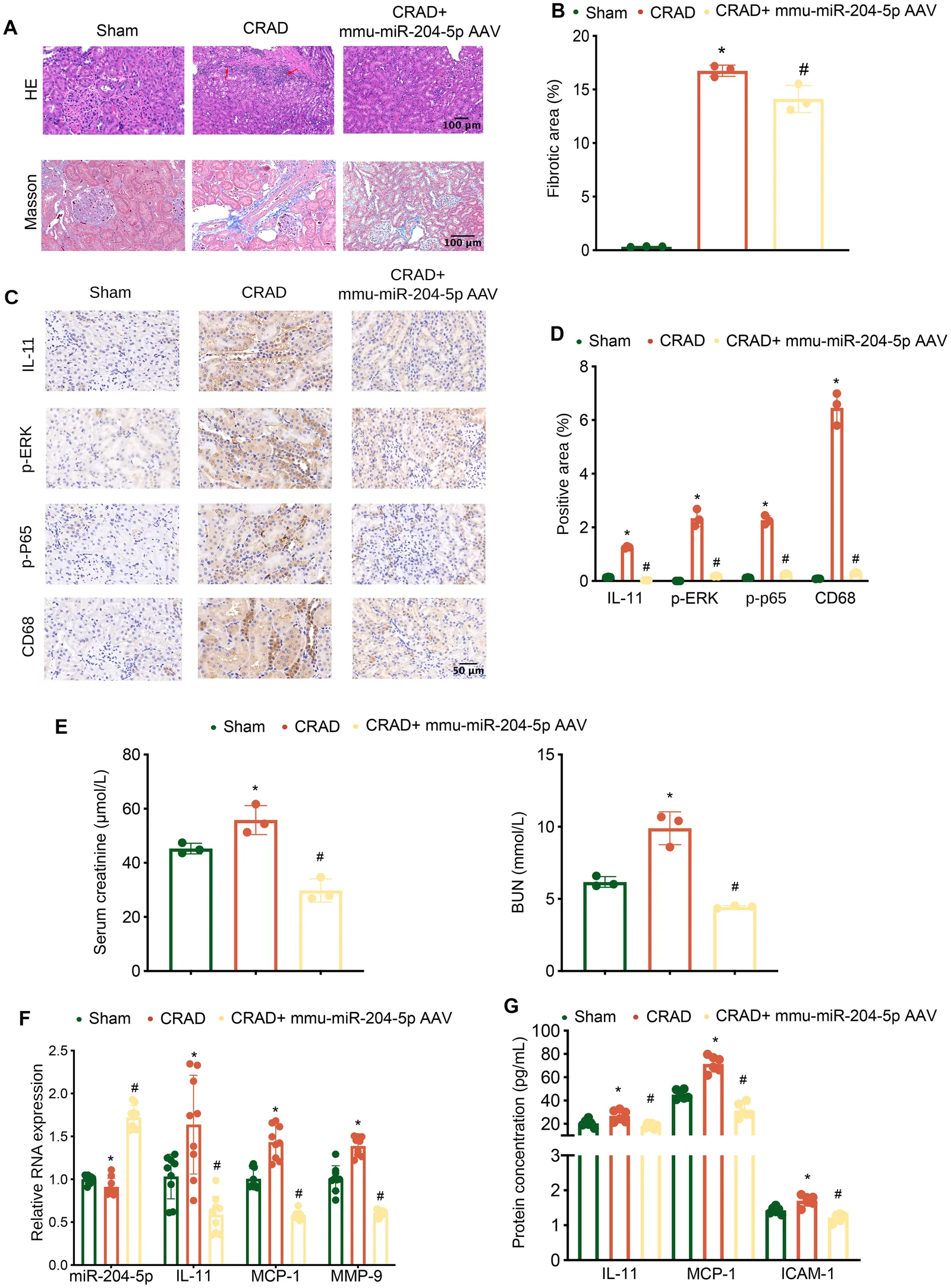
miR-204-5p overexpression attenuates renal inflammatory infiltration and IL-11/ERK1/2-NF-κB activation in early-stage CRAD. **(A)** Representative H&E and Masson’s trichrome staining images of kidney tissues from the Sham, CRAD, and CRAD + mmu-miR-204-5p AAV groups. H&E staining showed tubulointerstitial injury and inflammatory cell infiltration in CRAD kidneys, which were partially attenuated after mmu-miR-204-5p AAV treatment. Red arrows indicate representative inflammatory lesions. Scale bars, 100 μm. **(B)** Quantitative analysis of fibrotic area based on Masson’s trichrome staining. **(C)** Representative immunohistochemical staining images of IL-11, p-ERK, p-p65, and CD68 in kidney tissues. Scale bar, 50 μm. **(D)** Quantitative analysis of IL-11-, p-ERK-, p-p65-, and CD68-positive areas. **(E)** Serum creatinine and BUN levels in each group. **(F)** Relative RNA expression levels of miR-204-5p, IL-11, MCP-1, and MMP-9 in kidney tissues. **(G)** Protein concentrations of IL-11, MCP-1, and ICAM-1 in kidney tissues. The broken y-axis indicates different concentration ranges. Data are presented as mean ± SD; n = 3 rats per group. ^*^P < 0.05 vs. Sham group; ^#^*P* < 0.05 vs. CRAD group.

To further validate the protective role of miR-204-5p *in vivo*, an AAV vector carrying mmu-miR-204-5p was administered to CRAD rats. Histological analysis showed that CRAD kidneys exhibited prominent tubular-interstitial injury, inflammatory cell infiltration, and collagen deposition compared with the Sham group. In contrast, mmu-miR-204-5p AAV treatment alleviated these pathological changes, as shown by H&E and Masson’s trichrome staining (**Figure 8A**). Quantitative analysis further confirmed that the fibrotic area was significantly reduced in the CRAD + mmu-miR-204-5p AAV group compared with the CRAD group (**Figure 8B**).

We next examined whether miR-204-5p overexpression affected IL-11-associated inflammatory signaling in CRAD kidneys. Immunohistochemical staining showed that IL-11, p-ERK, p-p65, and CD68 were markedly increased in CRAD kidneys, indicating activation of IL-11-associated signaling and enhanced macrophage infiltration. Notably, mmu-miR-204-5p AAV treatment reduced IL-11 expression, suppressed p-ERK and p-p65 levels, and decreased CD68-positive macrophage accumulation in CRAD kidneys (**Figures 8C, D**).

Consistent with the improvement in renal histopathology, mmu-miR-204-5p AAV treatment significantly decreased serum creatinine and blood urea nitrogen levels in CRAD rats, suggesting improved renal function (**Figure 8E**). RT-qPCR analysis confirmed that mmu-miR-204-5p AAV increased miR-204-5p expression in CRAD kidneys. In parallel, IL-11, MCP-1, and MMP-9 RNA levels were significantly reduced after miR-204-5p overexpression (**Figure 8F**). Moreover, the protein levels of IL-11, MCP-1, and ICAM-1 were elevated in CRAD kidneys and were decreased following mmu-miR-204-5p AAV treatment (**Figure 8G**).

Collectively, these findings indicate that rmIL-11 partially rescues the inhibitory effects of miR-204-5p mimic on ERK1/2-NF-κB activation and inflammatory mediator expression *in vitro*. Furthermore, *in vivo* overexpression of miR-204-5p attenuates renal inflammatory infiltration, fibrotic remodeling, and functional impairment in early-stage CRAD, which is associated with suppression of IL-11/ERK1/2-NF-κB-related inflammatory signaling.

## 4 Discussion

Chronic renal allograft dysfunction (CRAD) remains a major barrier to long-term renal allograft survival. Interstitial fibrosis and tubular atrophy (IF/TA) are recognized as common pathological features of chronic graft injury, and inflammatory infiltration is closely associated with fibrotic remodeling and graft functional deterioration (2, 3). Although fibrosis is often considered as a final pathological outcome, inflammatory cell infiltration and inflammatory mediator production may actively participate in the initiation and progression of CRAD-related tissue injury (3). In the present study, kidney tissues were collected at 12 weeks post-transplantation. This time point was selected to capture an early stage of CRAD progression, when inflammatory infiltration, renal functional impairment, and early fibrotic remodeling were detectable but before the development of advanced irreversible fibrosis. Therefore, the 12-week time point provided an appropriate window to investigate early inflammatory events and their regulatory mechanisms during CRAD progression. This design is consistent with the focus of the present study on early inflammatory infiltration rather than end-stage graft fibrosis.

In this study, we identified a miR-204-5p/IL-11 regulatory axis that contributes to early inflammatory injury in CRAD. Early-stage CRAD kidneys showed marked IL-11 upregulation, accompanied by inflammatory cell infiltration, fibrotic remodeling, ERK1/2-NF-κB activation, and renal dysfunction. Inhibition of IL-11 by IL-11-shRNA AAV attenuated renal inflammatory infiltration and improved renal injury. *In vitro*, IL-11/IL-11RA signaling contributed to TGF-β1-induced inflammatory activation in BUMPT cells, whereas ERK1/2 inhibition suppressed NF-κB activation and inflammatory mediator expression. Mechanistically, miR-204-5p directly targeted the 3′-UTR of IL-11 mRNA and reduced IL-11 expression. Functional rescue with rmIL-11 partially restored ERK1/2-NF-κB activation and inflammatory mediator expression suppressed by miR-204-5p mimic. Furthermore, *in vivo* overexpression of miR-204-5p by mmu-miR-204-5p AAV alleviated IL-11-associated inflammatory signaling and renal injury in CRAD rats. Together, these findings place miR-204-5p upstream of the IL-11/ERK1/2-NF-κB inflammatory cascade in early CRAD.

IL-11 is a member of the IL-6 cytokine family and signals through a receptor complex involving IL-11RA and gp130 (23, 24). Increasing evidence indicates that IL-11 functions as an important fibro-inflammatory cytokine in multiple organs, including the kidney (5, 25, 26). Previous studies have shown that IL-11 can promote fibroblast activation, extracellular matrix deposition, epithelial injury responses, and tissue inflammation through noncanonical signaling pathways (5, 7, 25, 27). However, the role of IL-11 in CRAD-associated early inflammatory injury remains insufficiently defined. Our *in vivo* data showed that IL-11 was markedly increased in early-stage CRAD kidneys. More importantly, IL-11 knockdown by IL-11-shRNA AAV reduced CD68-positive macrophage accumulation, inflammatory mediator expression, fibrotic area, and renal dysfunction. These observations indicate that IL-11 is not merely a marker of CRAD-associated injury, but functionally contributes to renal inflammatory and fibrotic remodeling during early CRAD progression.

In the *in vitro* experiments, TGF-β1 stimulation increased IL-11 and IL-11RA expression in BUMPT cells, together with elevated expression and secretion of inflammatory mediators. Neutralization or siRNA-mediated knockdown of either IL-11 or IL-11RA attenuated these inflammatory responses, suggesting that IL-11/IL-11RA signaling participates in tubular epithelial inflammatory activation. Although these findings support the involvement of IL-11-associated signaling, the current data do not fully establish a definitive IL-11 autocrine feed-forward loop. Demonstrating such a loop would require additional dynamic evidence, including time-course analyses of IL-11 secretion, IL-11RA/gp130 signaling complex activation, and blockade of secreted IL-11 signaling. Therefore, we describe this mechanism more conservatively as IL-11/IL-11RA-associated inflammatory signaling. In addition, extensive studies have highlighted the pivotal role of the IL-11-ERK1/2 signaling pathway in renal and cardiac fibrosis (25). In our study, both CRAD kidney tissues and TGF-β1-stimulated BUMPT cells showed increased p-ERK and p-p65 levels. Inhibition of ERK1/2 by PD98059 reduced p-ERK, decreased p-p65, and suppressed inflammatory mediator expression both *in vivo* and *in vitro*. Moreover, rmIL-11 stimulation activated ERK1/2-NF-κB-related signaling in BUMPT cells, whereas PD98059 attenuated rmIL-11-induced inflammatory activation. These results suggest that ERK1/2 contributes to NF-κB activation and inflammatory mediator production downstream of IL-11-associated signaling. However, because IL-11 may also activate additional downstream pathways, the ERK1/2-NF-κB axis should be considered an important but not exclusive downstream mechanism of IL-11 in CRAD.

The choice of miR-204-5p was based on both biological plausibility and target prediction. miRNAs are well suited to regulate inflammatory and fibrotic programs because they can simultaneously tune multiple transcripts within related pathways (8–13, 28). Among renal disease-associated miRNAs, miR-204-5p has been described as a kidney-enriched protective molecule in chronic injury settings (29, 30). In our experiments, the predicted interaction between miR-204-5p and the IL-11 3′-UTR was confirmed by luciferase reporter assays, and miR-204-5p mimic reduced IL-11 expression in TGF-β1-exposed BUMPT cells. This regulatory relationship is not entirely unprecedented, as previous studies have reported that miR-204-5p can directly target IL-11 in esophageal squamous cell carcinoma and that a DGUOK-AS1/miR-204-5p/IL-11 axis contributes to breast cancer progression. Therefore, the novelty of the present study does not lie in identifying IL-11 as a general target of miR-204-5p, but rather in defining the relevance of the miR-204-5p/IL-11 axis to early inflammatory injury and IL-11-associated ERK1/2-NF-κB signaling in CRAD. The rescue experiment provided functional support for this regulatory relationship: exogenous rmIL-11 partially restored ERK1/2-NF-κB activation and increased MCP-1 and MMP-9 production despite miR-204-5p mimic transfection. The incomplete rescue is also informative, as it implies that miR-204-5p may regulate IL-11-dependent and IL-11-independent inflammatory mechanisms in parallel.

TGF-β signaling is involved in a wide range of fibrotic processes and plays an important role in renal fibrotic remodeling and epithelial injury responses (31, 32). In this study, TGF-β1-stimulated BUMPT cells were used as an *in vitro* model to mimic CRAD-associated early inflammatory and pro-fibrotic responses in renal tubular epithelial cells. This model cannot fully reproduce the complex immune, vascular, and hemodynamic microenvironment of renal allografts; however, it provides a controlled system to investigate tubular epithelial cell-intrinsic signaling mechanisms under a defined pro-inflammatory and pro-fibrotic stimulus. In our study, TGF-β1 stimulation increased IL-11, IL-11RA, and inflammatory mediators, supporting the suitability of this model for studying the miR-204-5p/IL-11 axis in CRAD-related tubular epithelial inflammatory activation.

From a translational perspective, the miR-204-5p/IL-11 axis may provide a potential target for modulating early inflammatory injury in CRAD. Compared with broad immunosuppression, targeting a defined fibro-inflammatory signaling pathway may offer a more specific approach to limiting early inflammatory amplification and subsequent fibrotic remodeling. Existing studies have suggested that modulation of inflammatory, oxidative stress-related, ferroptosis-related, and chemokine-mediated pathways may attenuate CRAD progression (33–36). IL-11-targeted intervention may be particularly relevant because IL-11 is increasingly recognized as a fibro-inflammatory mediator in multiple chronic organ diseases (5, 25–27, 37–39). In addition, miR-204-5p may have potential value as an upstream regulatory molecule or biomarker reflecting IL-11-associated inflammatory activation. Nevertheless, translating miRNA-based strategies into clinical therapy remains challenging because of issues related to delivery efficiency, tissue specificity, stability, off-target effects, and long-term safety (28, 30).

This study has several limitations. The animal experiments were performed with a limited sample size, which may affect the generalizability of the findings. Human renal allograft samples were not available in the present work, so the clinical relevance of the miR-204-5p/IL-11 axis remains to be verified in patient tissues. Another limitation is the lack of matched scrambled AAV control groups for the *in vivo* IL-11 knockdown and miR-204-5p overexpression experiments; therefore, vector-related or sequence-independent effects cannot be fully excluded. In addition, the TGF-β1-stimulated BUMPT cell model captures only epithelial cell-intrinsic responses and cannot model immune-cell crosstalk within the allograft microenvironment. Finally, although the data support IL-11/IL-11RA-associated signaling, further studies involving time-course experiments, gp130 pathway interrogation, and receptor-blocking approaches are needed to define the dynamics of IL-11 signaling more precisely.

In summary, this study proposes that miR-204-5p acts as an upstream brake on IL-11-associated inflammatory signaling during early CRAD. By targeting the IL-11 3′-UTR, miR-204-5p reduces IL-11 expression and limits ERK1/2-NF-κB-related inflammatory activation. AAV-mediated miR-204-5p overexpression *in vivo* further attenuated macrophage infiltration, fibrotic remodeling, and renal dysfunction in CRAD rats. These results provide mechanistic support for the miR-204-5p/IL-11 axis as a regulator of early inflammatory injury after renal transplantation.

## Data Availability

The original contributions presented in the study are included in the article. Further inquiries can be directed to the corresponding authors.
